# Case report: A unique presentation of a high-grade neuroepithelial tumor with EWSR1::PATZ1 fusion with diagnostic, molecular, and therapeutic insights

**DOI:** 10.3389/fonc.2023.1094274

**Published:** 2023-01-31

**Authors:** Andre Ene, Jing Di, Janna H. Neltner, Thomas Pittman, Susanne M. Arnold, Jill M. Kolesar, John L. Villano, Sara E. Bachert, Derek B. Allison

**Affiliations:** ^1^ Department of Pathology and Laboratory Medicine, University of Kentucky, Lexington, KY, United States; ^2^ Department of Neurosurgery, University of Kentucky, Lexington, KY, United States; ^3^ Department of Medicine, Division of Medical Oncology, University of Kentucky, Lexington, KY, United States; ^4^ Markey Cancer Center, University of Kentucky, Lexington, KY, United States; ^5^ Department of Pharmacy Practice and Science, University of Kentucky, Lexington, KY, United States

**Keywords:** brain tumor, neurooncology, pediatric, neuroepithelial, PATZ1, EWSR1, gene fusion

## Abstract

**Background:**

EWSR1::PATZ1 fusion tumors are exceedingly rare in the central nervous system with only 14 prior cases documented. PATZ1 fusion neuroepithelial tumors are beginning to be recognized as a distinct molecular class of neoplasms that most often occur in children and young adults. These tumors are polyphenotypic, show diverse morphologic features, may be low- or high-grade, and tend to have an intermediate prognosis.

**Case presentation:**

Herein, we present an unusual case of a high-grade neuroepithelial tumor in a young man with an EWSR1::PATZ1 fusion. This case is unique because the tumor appears to have undergone high-grade transformation from a persistent low-grade glioma, which has yet to be reported. Furthermore, this case is the first to document concurrent RB1 loss, SMAD4 loss, and TP53 inactivation in this tumor type, which correlates with high-grade transformation. Fortunately, this patient is alive 2.5 years after treatment and 18.5 years after initial presentation, which provides a unique window into how these tumors clinically behave over a long follow-up period. Finally, we discuss the altered molecular pathways that are a result of the EWSR1::PATZ1 fusion and discuss potential therapeutic targets.

**Conclusion:**

Awareness of the emerging entity of PATZ1 fusion neuroepithelial tumors is important not only for accurate diagnostic and prognostic purposes but also for predicting response to therapy.

## Introduction

The histological diagnosis of central nervous system (CNS) tumors is challenging because many share similar morphological features. Until recently, CNS tumors have been diagnosed on histological findings and ancillary testing such as immunohistochemistry; however, specific molecular testing is now standard of care for many tumors and is providing important diagnostic and prognostic information. The recent 2021 WHO classification of CNS tumors reflects this shift toward molecular diagnostics (1). There are many well-known, recurrent genetic alterations in CNS tumors, such as 1p19q codeletions of oligodendrogliomas, loss of ATRX in astrocytomas (2), and SLC44A1::PRKCA gene fusions in papillary glioneuronal tumors (3). Recently, a small subset of CNS neuroepithelial tumors have been identified with an EWSR1::PATZ1 gene fusion (3). The Ewing sarcoma breakpoint region 1 (EWSR1) is one of the most vulnerable genes to breakage and translocation (4) and has been documented in a number of malignant neoplasms. EWSR1 has a number of fusion partners; however, the EWSR1::PATZ1 fusion has also recently been described in a rare subset of sarcomas that show variable morphologic features, immunoprofiles, and clinical behavior (5, 6). To date, only 14 neuroepithelial tumors with an EWSR1::PATZ1 fusion have been reported in the literature. From this data, the tumors most often occur in children and young adults, are polyphenotypic, may show diverse morphologic features, may be low- or high-grade, and tend to have an intermediate prognosis. However, since the first reports in 2017, many of the cases referenced in the literature lack thorough clinicopathologic descriptions and details. Importantly, recent data suggests that increased PATZ1 inhibits TRAIL-mediated apoptosis and that PATZ1 fusion neuroepithelial tumors may have some intrinsic resistance to temozolomide, which functions through the TRAIL pathway. As a result, accurately identifying these cases may have important therapeutic implications (7). Herein, we present a CNS tumor with an EWSR1::PATZ1 gene fusion, a unique clinical course, and co-existing previously undescribed genetic alterations in this tumor type.

## Case presentation

**Figure 1:**
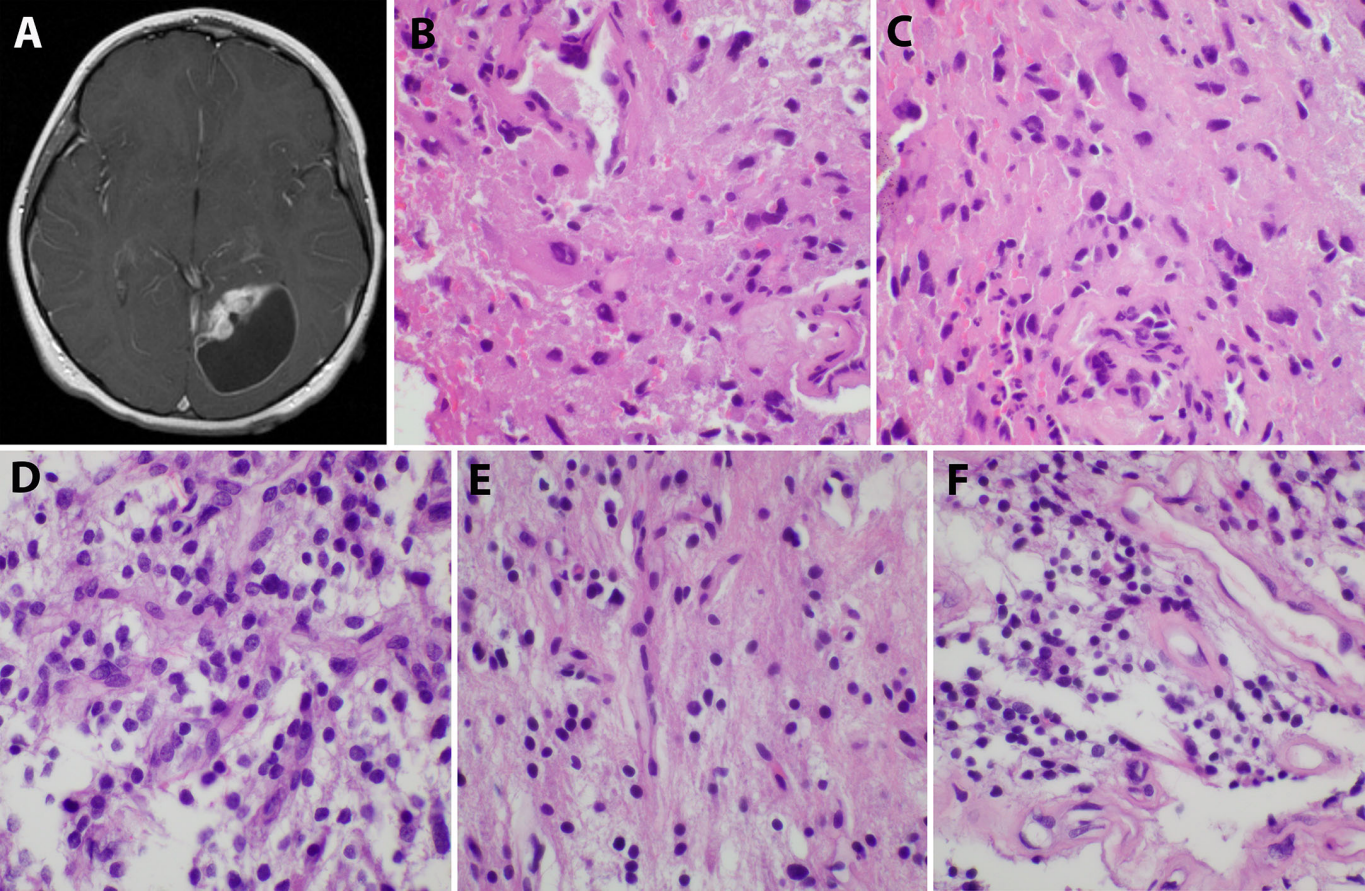
Initial imaging presentation and histological studies of the tumor. **(A)**. Imaging showed a cystic lesion with an enhancing mural nodule in the left occipital lobe; **(B, C)**. H&E staining of the initial tumor revealed a low-grade astrocytic proliferation with scattered hyperchromatic nuclei and eosinophilic cytoplasm; **(D–F)**. The second recurrent tumor displayed low-grade astrocytic features similar to the original tumor and some endothelial proliferation.

**Figure 2:**
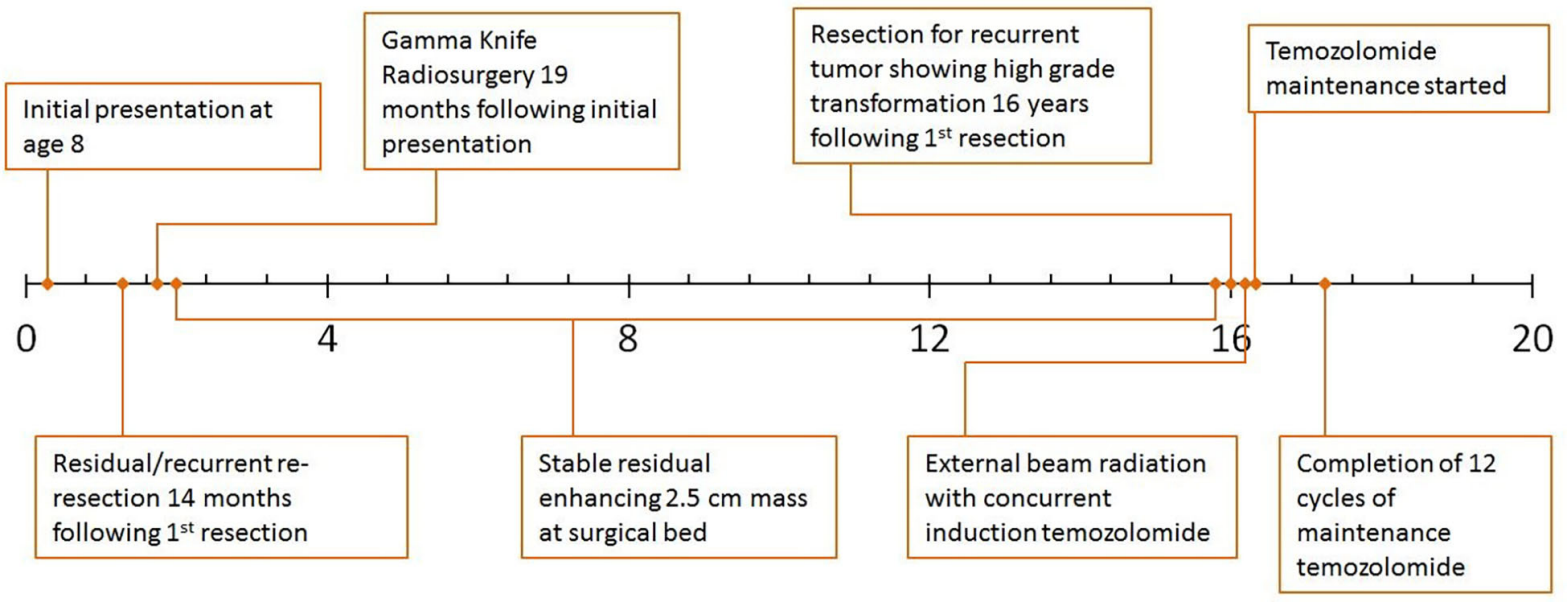
Clinical timeline.

An 8-year-old Caucasian male presented with a six-month history of increasingly severe headaches associated with physical exertion. Imaging studies revealed a cystic lesion with an enhancing mural nodule in the left occipital lobe (**Figure 1A**). The patient underwent a subtotal resection 18.5 years ago, tolerating the procedure well with no neurological deficits (see **Figure 2** for timeline). As shown in **Figure 1B**, the tumor was comprised of a low-grade astrocytic proliferation with scattered hyperchromatic nuclei. There was a small zone of necrosis and some endothelial proliferation; however, mitoses were not increased, and the MIB-1 index was low (less than 5%). On retrospective staining, the tumor was negative for BRAF V600E by immunohistochemistry. At the time, the tumor was thought to be most compatible with a pleomorphic xanthoastrocytoma (PXA), WHO grade 2 (**Figure 1B, C**). Post-operative imaging revealed a residual solid/cystic nodule of tumor (3 cm), which remained stable during subsequent follow-up scans until one year later, when he had symptomatic enlargement of the mass (5.7 x 4.7 cm). At this time, he underwent a second resection. The tumor continued to show low-grade astrocytic features and was morphologically compatible with recurrent PXA (**Figure 1D–F**). Again, the tumor was retrospectively stained for BRAF V600E, which continued to be negative. Several months later, he developed headaches again and an MRI showed a second recurrence at the site of prior resection. He underwent Gamma knife radiosurgery (18 Gy), which resolved his symptoms. Repeated imaging over the next 9 years showed continued but stable nodular enhancement (2.4-2.5 cm) within the tumor resection bed.

**Figure 3:**
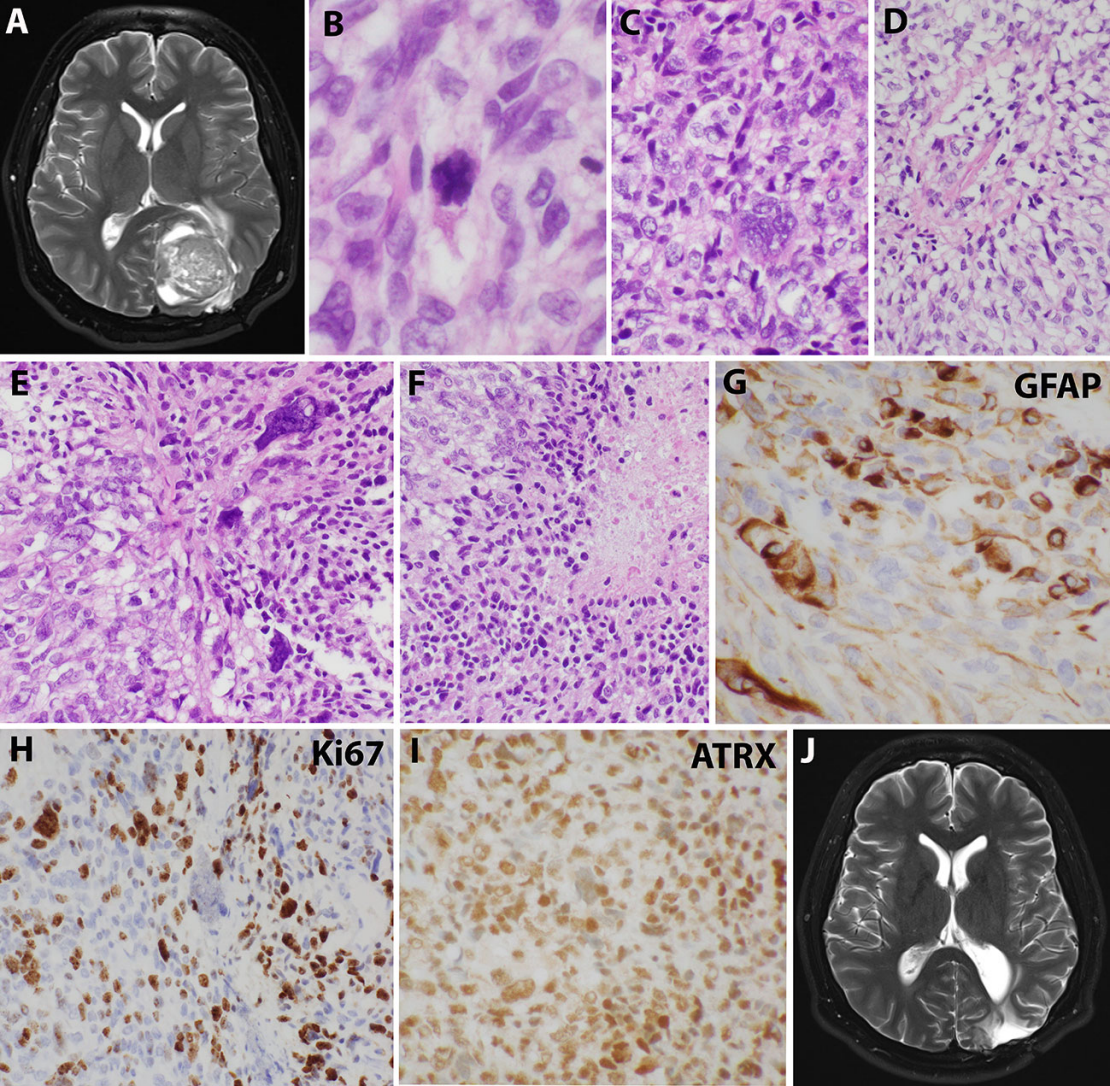
Imaging and microscopic studies of the newly recurrent tumor. **(A)**, Imaging revealed a 6 cm irregular, contrast-enhancing mass with solid and cystic components associated with the prior tumor site causing significant mass effect.**(B–F)**, H&E staining showed heterogeneous and poorly differentiated tumor cells with atypical mitotic figures **(B)**, numerous bizarre multinucleated giant cells **(C)**, clear cells change with eccentrical nuclei **(D)**, fusiform and pleomorphic monstrous nuclei **(E)**, and foci of palisading necrosis with tumor cells lining the ischemic edges **(F)**. **(G)**, Pleomorphic tumor cells were positive for GFAP staining. **(H)**, Ki67 stain showed high mitotic index. **(I)**, ATRX stain was retained. **(J)**, Postoperative imaging demonstrated stable enhancing foci in the surgical bed.

Sixteen years after his initial presentation, he returned with severe headaches and a right visual field deficit. Imaging revealed a 6 cm irregular, contrast-enhancing mass with solid and cystic components associated with the prior tumor site causing significant mass effect (**Figure 3A**). A third resection was performed, showing a hypercellular glial neoplasm with marked atypia, palisading necrosis, and increased and atypical mitoses. (**Figure 3B–F**). Immunohistochemical stains were positive for GFAP (**Figure 3G**) and the MIB-1 index was approximately 50% (**Figure 3H**). Stains for BRAF V600E and IDH1 R132H were negative and ATRX was intact (**Figure 3I**). Overall, the findings were most consistent with a glioblastoma, IDH-wildtype, WHO grade 4. Additional genetic testing was performed using the FoundationOne ^®^CDx panel, which showed an EWSR1 (NM_005243)-PATZ1(NM_014323) fusion, RB1 loss, SMAD4 loss, and a TP53 L330fs*15 inactivating mutation. The tumor was microsatellite stable and showed a low mutational burden (0 Muts/Mb). The patient received 6 weeks of external beam radiation with a total of 60 Gy along with temozolomide induction at 200 mg/day for 28 days. The patient then received 12 cycles of maintenance temozolomide therapy (cycle 1 at 150 mg/m (2) day with dose-escalation for cycles 2-12 at 200 mg/m (2) day). On clinical follow-up, the patient has done well with no new symptoms, and MRI has demonstrated stable enhancing foci in the surgical bed with no new abnormal enhancing foci on repeated imaging over the last 2.5 years since surgery (**Figure 3J**).

## Discussion

Our case presentation is unique for several reasons. One, we provide a detailed clinical history and pathologic review of a low-grade glial tumor that persisted and eventually underwent high-grade transformation. This tumor was shown to have an EWSR1::PATZ1 fusion and represents the first documented transformed tumor of this type. Next, this tumor showed molecular evidence of this high-grade transformation with co-existing loss of RB1 and SMAD4, as well as a TP53 mutation—none of which have been documented in prior neuroepithelial tumors with EWSR1::PATZ1 fusion. Finally, our patient is alive and well 18.5 years after initial diagnosis and 2.5 years after high-grade transformation. This presentation provides a unique window into how these tumors clinically behave over a longer period of follow-up than previous reports.

EWSR1 gene is found on chromosome 22q12.2, spans 40 kb, consists of 17 exons, and is part of the Ten Eleven transcription factor (TET) family of RNA-binding proteins (4). The gene product is a protein involved in DNA recombination and repair, mRNA splicing, G-protein signaling, and mitotic regulation (4). In normal neuronal cells, EWSR1 is required for cell survival (4). PATZ1, a transcription factor, belongs to the BTB-ZF (broad-complex, tramtrack and bric-à-brac -zinc finger) family and is also found on chromosome 22q12.2, just two 2 MB away from the EWSR1 gene (3, 4, 8). It is involved in DNA recognition, RNA packaging, transcriptional activation, protein folding and assembly, and the regulation of apoptosis (4). Importantly, PATZ1 is a regulator of embryonal stem cell pluripotency, especially in neural differentiation, which is achieved by repressing developmental genes through its BTB domain (2). Fusions of EWSR1 and PATZ1 are a result of an inversion of chromosome 22, involving exon 8 of EWSR1 and the zinc finger region of exon 1 in PATZ1 (4). The N-terminal transcriptional activation domain of EWSR1 combines with the C-terminal DNA binding domain of PATZ1, resulting in the removal of the N-terminal repressor domain of PATZ1, which allows for aberrant expression and altered transcriptional signatures (2, 3). These fusions can be detected using whole genome or exome sequencing or RNA sequencing. Identifying this fusion by fluorescence *in-situ* hybridization (FISH) can be technically challenging because the genes are very close together, making it difficult to interpret and distinguish the red and green break-apart signals (2).

In addition to the EWSR1::PATZ1 fusion, an inactivating TP53 mutation was present, along with loss of RB1 and SMAD4. RB1 is a tumor suppressor gene and is a negative regulator of the G1/S checkpoint of the cell cycle. In the Cancer Genome Atlas (TCGA) cohort, homozygous deletion or RB1 mutation was identified in roughly 3% and 10% of low-grade and high-grade gliomas, respectively, but has not been specifically identified in any of the prior EWSR1:PATZ1 fusion neuroepithelial tumors (9). Inactivation of RB1 is more common in secondary glioblastomas, but there isn’t clear data that this inactivation is a reliable independent predictor of survival (10–13). In addition, SMAD4 is a tumor suppressor regulating transcriptional activity downstream of TGFβ receptor. Inactivating mutations or homozygous deletion in SMAD4 is commonly found in gastrointestinal cancers, most frequently in pancreatic adenocarcinomas but also less frequently in cholangiocarcinomas and adenocarcinomas of the appendix and colon, to name a few (14–19). Decreased expression of SMAD4 in these tumor types, as well as others such as breast and prostate cancers, has been associated with worse overall survival (20, 21). Likewise, decreased SMAD4 IHC protein expression and mRNA levels have been associated with poor outcomes in patients with gliomas (22). Like RB1, SMAD4 loss has not been reported in any of the prior EWSR1::PATZ1 fusion neuroepithelial tumors. TP53 is another tumor suppressor gene, where alterations are found in 35% of glioblastomas, with a higher incidence in secondary and pediatric glioblastomas (18, 23). Interestingly, TP53 mutation is correlated with a favorable prognosis in glioblastomas, which often are present in lower grade astrocytomas that undergo high-grade transformation after developing coexisting loss of CDKN2A, RB1, or PTEN—not unlike the current case (24). Like the loss of RB1 and SMAD4, TP53 mutations have not been reported in clinical samples of prior EWSR1-PATZ1 fusion neuroepithelial tumors.

While not done in our case, methylations profiles have been shown to aid in tumor classification, as tumors maintain their epigenetic methylation of their cell of origin (3). Previous methylation profiles involving EWSR1::PATZ1 fusions have suggested a new entity based on distinct methylation clusters (2). Additionally, recurrent copy number variations of chromosome 22 in EWSR1::PATZ1 fusions support the idea of a novel molecular tumor type (3). This observation is further supported by the fact that these fusion CNS tumors have incredibly diverse clinical and histopathologic presentations. Of the 14 documented cases of EWSR1-PATZ1 fused CNS tumors, morphologic descriptions have ranged from showing round, monomorphic cells with clear cytoplasm, vascular hyalinization, and oligodendroglia-like features to markedly atypical with hyperchromatic and polymorphous nuclei, as well as other high-grade features. As a result, diagnoses have included glioblastoma, anaplastic ependymoma, primitive neuroendocrine tumor, pleomorphic xanthoastrocytoma, high- and low-grade gliomas, BRAF V600E negative ganglioglioma, papillary glioneuronal tumor, undifferentiated CNS sarcoma and malignant neuroepithelial tumor with sarcomatous differentiation.

When taken together, the neuroepithelial EWSR1::PATZ1 fusion tumors reported in the literature in children and young adults have no sex predilection, and the average age of onset is 20.4 years. Data suggests that these tumors behave clinically as intermediate grade; however, it is unclear how co-existing mutations in well-defined genes such as p53, SMAD4, and RB1 fit into the context of outcome prediction, given the paucity of data. Alhalabi et al. (3) suggest that these CNS tumors with PATZ1 fusions should be defined as a new molecular class of histologically diverse neuroepithelial tumors, referred to as “neuroepithelial tumor with PATZ1 fusion.”

In our case, the high-grade neuroepithelial tumor with EWSR1-PATZ1 fusion arose from a lower grade and recurrent tumor somewhat resembling a pleomorphic xanthoastrocytomas and fit previously reported descriptions of EWSR1-PATZ1 fusion cases with lower grade features. Interestingly, pleomorphic xanthoastrocytomas have been known to transform into anaplastic pleomorphic xanthoastrocytomas or glioblastomas years after initial diagnosis, as described by Watanabe et al (25). In our case, residual tumor was recurrent/present for 15 years after the initial diagnosis. Most likely, this tumor transformed after undergoing secondary hits to TP53, SMAD4, and RB1. High-grade transformation and these mutations have not been previously reported in neuroepithelial tumors with PATZ1 fusion. In the present case, the patient is doing well after presenting with high-grade transformation 2.5 years earlier. Despite no specific morphologic feature indicating this tumor represents anything other than a typical glioblastoma, the presence of the EWSR1::PATZ1 fusion and the rare reports in the literature make the case for a new molecular-based entity that requires additional investigation. This example further highlights the remarkable heterogeneity in these new fusion tumors, and the need for broad-spectrum molecular testing to help distinguish and better characterize neuroepithelial tumors with EWSR1::PATZ1 fusions.

One of the consistent challenges of treating gliomas is the identification of novel targets that are responsible for tumor recurrence and progression. One potential target for therapeutics appears to be PATZ1. PATZ1 is a prognostic factor in diffuse large B cell lymphomas, renal cell carcinoma, and serous ovarian tumors (26). Furthermore, PATZ1 knockout mice have been shown to develop several tumors, including non-Hodgkin lymphomas, sarcomas, and hepatocellular carcinoma (27). PATZ1 has also been shown to be upregulated in gliomas compared to normal brains, where high levels of PATZ1 expression is associated with high grade gliomas compared to low grade gliomas (28, 29). PATZ1 is highly expressed in adult glioblastomas (GBM) and is associated with higher expression in the proneural subtype, which has a longer survival than the mesenchymal subtype of glioblastoma (28). In the proneural subtypes, PATZ1 is preferentially expressed with glioma stem cells, which may explain why the proneural subtype of GBM resists standard therapy (28). PATZ1 has also been shown to decrease CXCR4 expression in GBM, which restricts differentiation of proneural GBMs to mesenchymal subgroup of GBMs (28). Studies have shown that (29) lower levels of expression of PATZ1 are also associated with poor outcomes in glioblastoma, including lower overall survival and progression-free survival (28).

Interestingly, in GBMs, PATZ1 can either be a tumor suppressor or an oncogene, depending on the cellular context (28). Interacting with TP53, PATZ1 binds P53-dependent gene promoters including Bax, CDKN1A, and MDM2, whereas in PATZ1 knockout models, the expression of Bax, CDKN1A and MDM2 are inhibited, and, as a result, the cells were resistant to apoptosis (27). However, in TP53-null cells, PATZ1 was found to downregulate the expression of Bax, CDKN1A, and MDM2, suggesting an oncogenic role of PATZ1 (27). Of note, our case also showed a TP53 mutation. In the presence of TP53, PATZ1 has been shown to upregulate PUMA, an apoptosis regulator of the Bcl-2 family that is a downstream target of TP53 (26). It causes mitochondria permeability, cytochrome C release, and apoptosis by binding to other Bcl-2 families (Bax, Bcl-2, Bcl-xl) (26). When PATZ1 interacts with PUMA, it leads to activation of intrinsic apoptotic pathways in glioblastomas (26). PATZ1 also promotes expression of other apoptosis related genes such as PARP1, caspase 3, capsase9, and Bax. In PUMA knockout models, the pro-apoptotic effects of PATZ1 were decreased even with overexpression of PATZ1 (27). Using siRNA, PATZ1 can be downregulated in glioma cells, resulting in upregulation of six pro-apoptotic genes linked to death receptor apoptosis; RIP, DAP-kinase 2, FADD, caspse8, Fas receptor, and DR5. Upregulation of these genes resulted in decreased resistance to both Fas-L and TRAIL-mediated apoptosis, in glioblastomas normally resistant to chemotherapy (7). This observation suggests that increased PATZ1 inhibits TRAIL-mediated apoptosis and that PATZ1 fusion neuroepithelial tumors may have some intrinsic resistance to temozolomide, which functions through the TRAIL pathway. Even though our patient was treated with temozolomide, a good surgical resection was performed, and external beam radiation was administered, and he is alive and well 2.5 years after high-grade transformation.

In summary, PATZ1 fusion neuroepithelial tumors appear to represent a distinct class of neoplasms with intermediate outcomes. Our case is unique in the literature in that the tumor appears to have undergone high-grade transformation. We also present a detailed clinical history, pathologic review, and molecular discussion to put the case into context. Finally, our patient is still alive 2.5 years after treatment for high-grade transformation and 18.5 years after initial presentation, which provides a unique window into how these tumors clinically behave over a long period of follow-up. Interestingly, PATZ1 may be more than just a diagnostic marker. PATZ1 is a useful marker of prognosis and is a strong contender as a potential therapeutic marker for GBM and other malignancies, due to both its roles in tumor suppression and oncogenesis. Furthermore, PATZ1 fusion neuroepithelial tumors may have some resistance to temozolomide through inhibition of TRAIL-mediated apoptosis. As a result, identifying additional therapeutic options is important. Fortunately, many genes also appear to be valid options for targeted therapeutics due to their close intrinsic interactions with PATZ1. Such potential molecular targets include DR5, rescuing TRAIL –induced apoptosis, PUMA, members of the Bcl-2 family, Fas-L, RIP, DAP-kinase 2, FADD, and caspase 8. However, given the rarity, proper identification of PATZ1 fusion neuroepithelial tumors is paramount to growing our understanding of their biology and clinical behavior.

## Data availability statement

The original contributions presented in the study are included in the article/supplementary material. Further inquiries can be directed to the corresponding author.

## Ethics statement

Ethical review and approval was not required for this retrospective study on a human participant in accordance with the local legislation and institutional requirements. Written informed consent to participate in this study was provided by the patient. Written informed consent was obtained from the individual(s) for the publication of any potentially identifiable images or data included in this article.

## Author contributions

DA, SA, JK, and JV contributed to the conception of the project. AE, JD, JN, and SB contributed to the figures, AE and JD wrote the first draft of the manuscript. DA and SB wrote sections of the manuscript. All authors contributed to the article and approved the submitted version.
